# Productivity of mother pigs is lower in countries that still confine them in gestation crates

**DOI:** 10.12688/f1000research.122042.1

**Published:** 2022-05-24

**Authors:** Cynthia Schuck-Paim, Wladimir J. Alonso

**Affiliations:** 1 Center for Welfare Metrics, Sao Paulo, Sao Paulo, 04795-100, Brazil

**Keywords:** pig, sows, gestation crates, confinement, animal welfare

## Abstract

**Background:** For decades, pig farmers have used gestation crates — small metal enclosures about two feet wide — to confine pregnant sows (female breeding pigs). Gestation crates physically restrain sows for most of their life, preventing them from walking or even turning around. Millions of females are still housed in these systems. Growing societal concern about animal welfare has been pressuring the industry for change, with recent legislation in the European Union and California restricting the use of crates. Still, the notion that gestation crates negatively affect sow welfare has been challenged by producers in regions where crates are widely used, who argue that, by facilitating health monitoring and preventing aggression, crates lead to lower sow mortality and higher piglet outputs per sow. We address these claims by comparing sow mortality and performance across countries with different housing systems.

**Methods:** To this end, we use publicly available data from InterPig, a network of pig production economists in 17 countries that provides internationally harmonized methods for meaningful comparisons of national production data.

**Results:** The results show that sow mortality is significantly higher, and annual pig production per sow significantly lower, in those countries where gestation crates are still the norm compared to countries in the European Union, where use of gestation crates is restricted to up to four weeks after insemination.

**Conclusions:** Claims of higher mortality and reduced productivity per sow in crate-free systems are not substantiated by this data. This evidence should be considered in policies affecting the welfare of breeding pigs.

## Introduction

**Figure 1.**
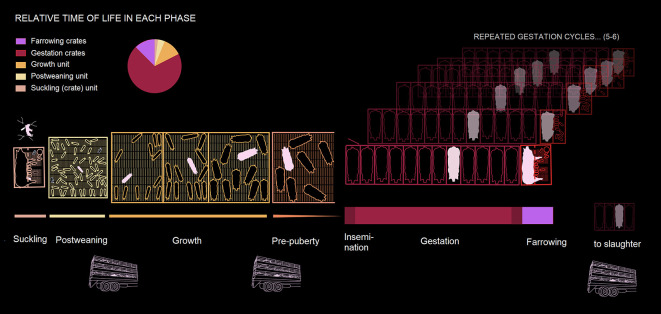
Life phases of a typical female breeding pig (pink) in conventional housing systems. Phases are ordered horizontally, from left to right, representing the passage of time. Except for the gestation and farrowing cycles (which are experienced five to six times by an average sow), enclosure widths roughly coincide with the duration of the corresponding phase. The thickness of lines underneath production phases is proportional to the time of life spent at each phase.

For decades, pig production has relied on the use of gestation crates (also referred to as gestation stalls) — small metal enclosures about two feet wide — to confine pregnant sows (female breeding pigs). Gestation crates physically restrain sows for most of their life, preventing them from walking, turning around or extending their limbs fully ^1^ ( Figure 1). They are linked to several welfare and health problems, such as pressure sores, ulcers, and abrasions, poorer cardiac function and immune-competence. ^2^^–^^5^ Most female breeding pigs around the globe are still housed in these systems.

However, growing societal concern about animal welfare ^6^^,^^7^ has been pressuring the industry for change. For example, with over 1 million European Union (EU) citizens supporting the EU citizens’ initiative ‘End the Cage Age’, the European Commission committed to present legislative proposals to prohibit the confinement of female pigs in gestation crates at any moment of their lives. ^8^ In California, similar legislation only allows confinement in enclosures providing a minimum of 24 square feet of usable floorspace per breeding pig. ^9^

Still, the notion that gestation crates negatively affect sow welfare is often challenged in countries and regions where crates are still widely used. The industry argues that, by facilitating health monitoring and preventing aggression, crates lead to lower sow mortality and higher piglet outputs per sow. ^10^ For example, according to the National Pork Producers Council (USA), crate-free housing “increases sow mortality, reduces litter sizes, and reduces productivity”. ^10^

Although mortality and productivity are not necessarily good indicators of welfare (sick individuals may be kept alive for a long time), ^11^ we explore these claims by comparing sow mortality and performance across countries in which different housing systems are used.

## Methods

We use publicly available data from InterPig, a network of pig production economists in 17 countries that provides internationally harmonized methods for meaningful comparisons of national production costs and performance indicators. ^12^^,^^13^ InterPig data are widely used by stakeholders in the swine industry, enabling assessment and comparison of sow productivity and mortality among countries with different policies regarding the housing of gestating pigs with an industry-validated dataset.

We analyzed sow mortality per year and number of pigs sold annually per sow. The latter parameter is very informative of sow productivity, being compounded by several factors: pigs born alive per litter, litters per sow per year and cumulative mortality of pigs over the production cycle (pre-weaning, rearing and finishing mortality). We used the last five years (2015–2019) of data on annual sow mortality and number of pigs sold annually per sow, as made available in the annual reports of the Agriculture and Horticulture Development Board (AHDB) and the Brazilian Agricultural Research Corporation (EMBRAPA). Data was used as provided in the reports, with no data points excluded. The underlying data is available at the Open Science Framework repository. ^14^

Countries were grouped in three housing categories: (1) countries where gestation crates for housing sows are still the norm (United States, Canada, Brazil), (2) countries where gestation crates are restricted to (up to) the first four weeks of pregnancy (Austria, Belgium, Czech Republic, Denmark, Finland, France, Germany, Hungary, Ireland, Italy, Netherlands, Spain) following a 2013 EU Directive, and (3) countries where gestation crates are entirely banned (Sweden and United Kingdom, where stalls were banned in 1994 and 1999, respectively).

We also investigated the extent to which potential differences in sow mortality and productivity among housing groups were statistically significant. To this end, we used a general linear model (GLM) having sow mortality and productivity as response variables, housing group as a fixed categorical variable and year as a co-variate. Group means were compared with Tukey’s post-hoc test. To standardize the distribution of residuals, sow productivity values were log-transformed and mortality data were square-root arcsine transformed. Analyses were conducted using Minitab v. 21.1.1. P-values are two-tailed.

## Results and conclusions

**Figure 2.**
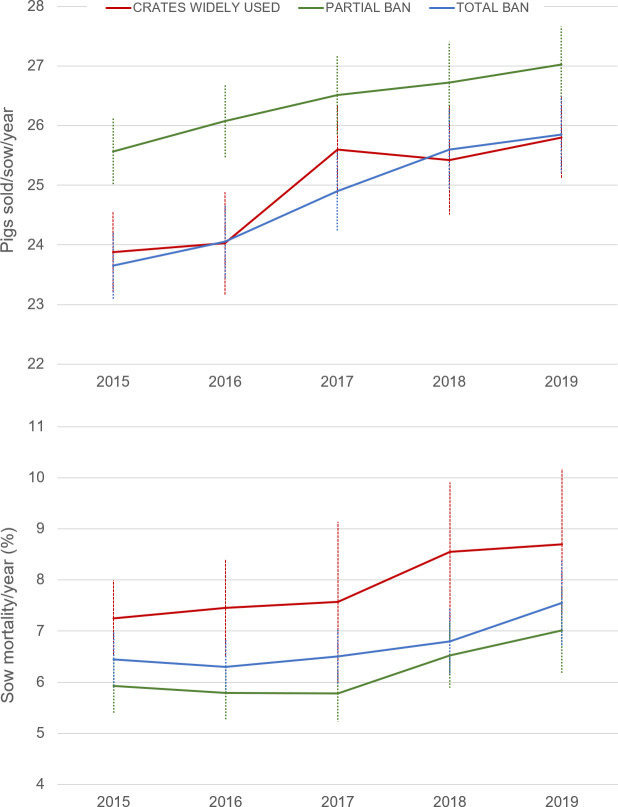
Average sow mortality (% year) and pigs sold/sow/year in three housing systems. Data from 17 countries belonging to the InterPig network, divided in three groups: (1) countries where gestation crates are the norm (Red: USA, Canada, Brazil), (2) gestation crates are restricted to (up to) the first four weeks of pregnancy (Black: Austria, Belgium, Czech Republic, Denmark, Finland, France, Germany, Hungary, Ireland, Italy, Netherlands, Spain), and (3) gestation crates are entirely banned (Blue: Sweden, United Kingdom (UK)). In the UK, data up to 2018 reflects a blend of indoor and free-range systems, and in 2019 indoor systems only. The patterns do not change if Brazil is removed from group 1.

Figure 2 shows mean values (± SEM) of sow productivity and mortality for each housing group, which have both increased over the five years (GLM, effect of year: *F*_1,85_=9.05, *P*=0.003 and *F*_1,85_=3.34, *P*=0.071, respectively). While many factors are expected to affect sow productivity and mortality, including the degree of commitment to national policies and legislation, Figure 2 clearly shows that sow mortality is not greater in crate-free systems. On the contrary, higher sow mortality is observed in those countries where gestation crates are still the norm (GLM: *F*_2,85_=5.06, *P*=0.009, effect of housing group) compared to those countries where crates have been restricted to four weeks after insemination (Tukey’s test, *P*=0.006). Likewise, there were significant differences in productivity among the housing groups (GLM: *F*_2,85_=5.99, *P*=0.004), with annual pig production per sow being significantly lower in countries where the use of gestation crates prevails compared to those where crates are restricted (Tukey’s test, *P*=0.012).

These results are in line with evidence showing that improving maternal welfare improves disease resistance, resilience and survival of piglets. ^3^^,^^4^^,^^15^ Importantly, they clearly speak against the notion that sow mortality is inherently higher, or productivity lower, in crate-free production. As observed in the transition of laying hens to cage-free systems, ^11^ variability in sow mortality might be observed during any transition from one housing system to another, though it is expected to decrease rapidly as farmers gain experience with the newly adopted systems. ^11^

Changes towards crate-free housing are currently underway in many countries and affect millions of pigs annually. The present findings should be considered to guide debate on policies and legislation affecting the welfare of breeding pigs.

## Data availability

### Underlying data

Open Science Framework: Productivity of mother pigs is lower in countries that still confine them in gestation crates. https://doi.org/10.17605/OSF.IO/G4DK2 ^14^

This project contains the following underlying data:

• DataSowMortalityProductivity.xlsx (Data on sow mortality and pigs sold per sow per year, from 2015 to 2019, for 17 countries in the InterPig Network)

Data are available under the terms of the Creative Commons Attribution 4.0 International license (CC-BY 4.0).
