# Productivity of mother pigs is lower, and mortality greater, in countries that still confine them in gestation crates

**DOI:** 10.12688/f1000research.122042.2

**Published:** 2022-08-04

**Authors:** Cynthia Schuck-Paim, Wladimir J. Alonso

**Affiliations:** 1 Center for Welfare Metrics, Sao Paulo, Sao Paulo, 04795-100, Brazil

**Keywords:** pig, sows, gestation crates, confinement, animal welfare

## Abstract

**Background**: For decades, pig farmers have used gestation crates to confine pregnant sows. Gestation crates physically restrain sows for most of their life, preventing them from walking or turning around. Growing concern about animal welfare has been pressuring the industry for change, with recent legislation in several countries restricting the use of crates. Still, the notion that gestation crates negatively affect sow welfare has been challenged by producers in regions where crates are still used, who argue that, by facilitating health monitoring and preventing aggression, crates lead to lower sow mortality and higher piglet outputs per sow. We test whether these claims are valid by comparing these parameters across countries with different housing systems.

**Methods**: We use publicly available data from InterPig, a network of pig production economists in 17 countries that provides harmonized methods for meaningful comparisons of production and cost indicators. We focus on the last five years (2015-2019) of data available. Annual sow mortality and the number of pigs sold per sow were compared among (1) countries where gestation crates are the norm (CRATE), (2) countries where gestation crates are restricted to four weeks after insemination (RESTRICTED), and (3) countries where gestation crates are banned (BANNED).

**Results**: Sow mortality was significantly higher (F _2,85_=5.03; *P*=0.009), and annual pig production per sow significantly lower (F _2,85_=5.99; *P*=0.004), in the CRATE than in the RESTRICTED group.

**Conclusions**: Claims of higher mortality and reduced productivity per sow in crate-free systems are not substantiated by this industry-validated dataset. While many factors differ among the country groups (e.g., genetics, nutrition, climate), the observation that factors other than crating have a greater influence on performance challenges claims of an overall negative effect of loose housing on the parameters investigated. This evidence should be considered in policies affecting the welfare of breeding pigs.

## Introduction

**Figure 1.**
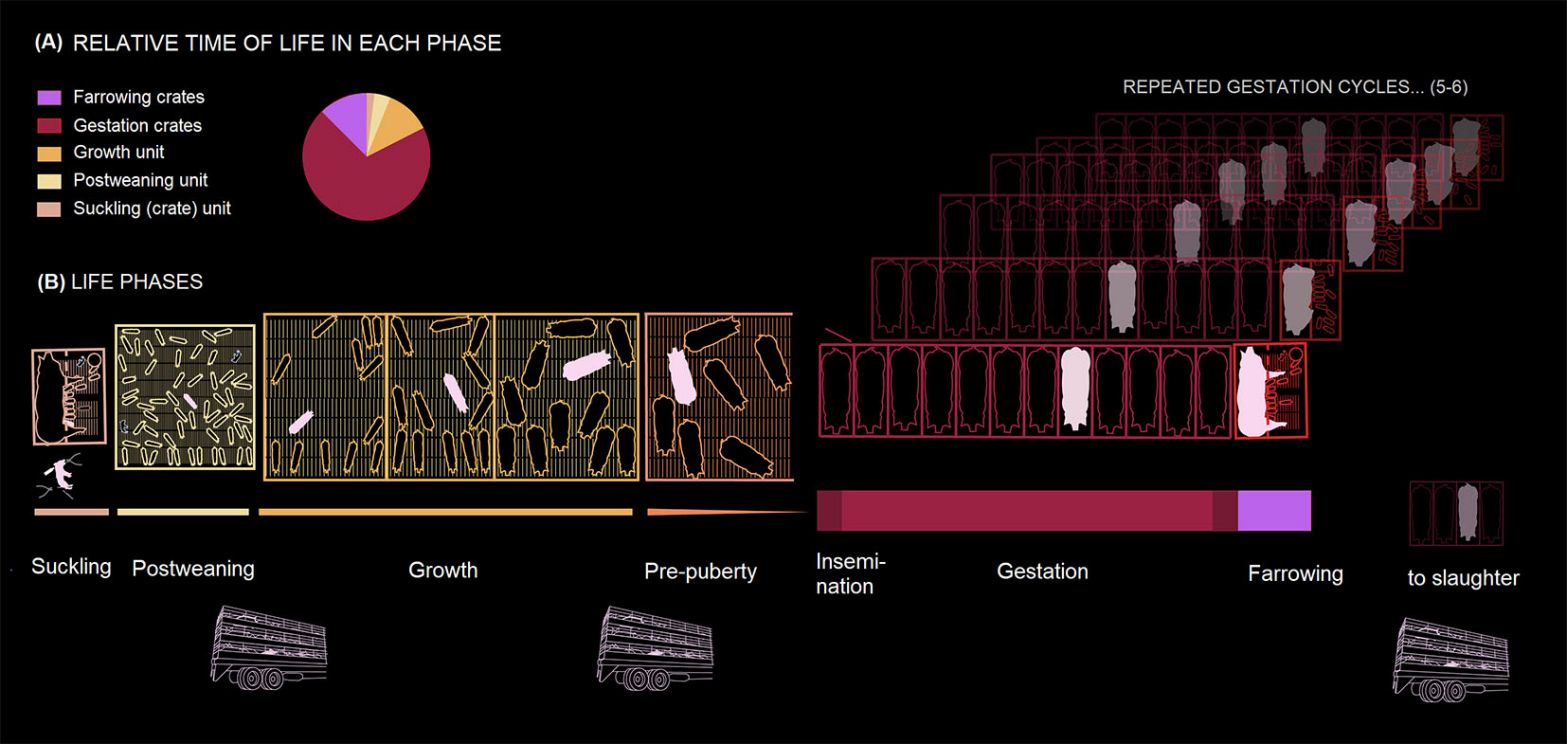
Life phases of a typical female breeding pig (pink) in conventional housing systems. (A) Relative time of life in each phase (pie chart), (B) Life phases are ordered horizontally, from left to right, representing the passage of time. Except for the gestation and farrowing cycles (which are experienced five to six times by an average sow), enclosure widths roughly coincide with the duration of the corresponding phase. The thickness of lines underneath production phases is proportional to the time of life spent at each phase.

For decades, pig production has relied on the use of gestation crates (also referred to as gestation stalls) — small metal enclosures about 0.6 by 2 m — to confine pregnant sows (female breeding pigs). Gestation crates physically restrain sows for most of their life, preventing them from walking, turning around or extending their limbs fully ^1^ ( Figure 1). They are linked to several welfare and health problems, such as pressure sores, ulcers, and abrasions, poorer cardiac function and immune-competence and a greater frequency of stereotypic behaviors. ^2^^–^^7^ Most female breeding pigs around the globe are still housed in these systems.

However, growing societal concern about animal welfare ^8^^,^^9^ has been pressuring the industry for change. For example, since 2013 use of gestation crates after four weeks of insemination has been banned in the European Union. In 2021, following the support of over 1 million European Union citizens for the EU citizens’ initiative ‘End the Cage Age’, the European Commission committed to present legislative proposals to prohibit the confinement of female pigs in gestation crates at any moment of their lives. ^10^ In California, similar legislation only allows confinement in enclosures providing a minimum of 24 square feet (2.2 square meters) of usable floorspace per breeding pig. ^11^

Still, the notion that gestation crates negatively affect sow welfare is often challenged in countries and regions where crates are still widely used. The industry argues that, by facilitating health monitoring and preventing aggression, crates lead to lower sow mortality and higher piglet outputs per sow. ^12^ For example, according to the National Pork Producers Council (USA), crate-free housing “increases sow mortality, reduces litter sizes, and reduces productivity”. ^12^ Similarly, the California Department of Food and Agriculture has considered that use of loose housing systems may lead to “lower piglet output per animal and increased breeding pig mortality”. ^11^

Although mortality and productivity are not necessarily good proxies of welfare, ^13^^,^^14^ in this Brief Report we explore whether these claims are factually valid by comparing the two parameters often cited – piglet output per sow and sow mortality – across countries in which different housing systems are used.

## Methods

We use publicly available data from InterPig, a network managed by the Agriculture and Horticulture Development Board (AHDB, a board of producers and other stakeholders in the UK farming industry), which collects farm and sector data from pig production economists in 17 countries that provides internationally harmonized methods for meaningful comparisons of national production indicators. Although InterPig data are used predominantly for cost comparisons across countries, this dataset also enables the assessment and comparison of sow performance and mortality among member countries with different policies regarding the housing of gestating pigs with an industry-validated dataset.

We analyzed the number of pigs sold annually per sow and sow mortality per year. The former parameter is very informative of sow productivity, being compounded by several factors: pigs born alive per litter, litters per sow per year and mortality of pigs over the production cycle [pigs sold/sow/year = pigs weaned/sow/year * ((100-rearing mortality)/100) * ((100-finishing mortality)/100), where pigs weaned/sow/year = pigs born alive per litter * litters/sow/year * ((100-pre-weaning mortality)/100)]. Importantly, the number of pigs sold annually per sow is of greater economic interest than the compounding factors cited, hence it is the parameter of choice in economic assessments of the impact of sow housing reforms. Sow mortality is represented by the percentage of sows that die on the farm during the year. The Interpig network does not provide estimates of sow culling rates.

To reflect the most recent statistics, we used the last five years of available data. Interpig data on the number of pigs sold per sow annually is described in the AHDB annual reports for the period 2015–2020. ^15^ Sow mortality statistics, as collected by the Interpig network, have been made publicly available in the reports of the Brazilian Agricultural Research Corporation (a member institution). ^16^ In the latter case, data for 2020 is only available for seven of the 17 countries. Thus, we restricted the analysis to the period 2015–2019. Data was used as provided in the reports, with no data points excluded. The underlying data is available at the Open Science Framework repository. ^17^

Countries were grouped in three housing categories: (1) countries where gestation crates for housing sows are still the norm (United States, Canada, Brazil) (CRATE group), (2) countries where gestation crates are restricted to (up to) the first four weeks of pregnancy (Austria, Belgium, Czech Republic, Denmark, Finland, France, Germany, Hungary, Ireland, Italy, Netherlands, Spain) following a 2013 EU Directive (RESTRICTED group), and (3) countries where gestation crates are entirely banned (Sweden and United Kingdom, where stalls were banned in 1994 and 1999, respectively) (BANNED group).

To investigate the extent to which potential differences in sow mortality and productivity among housing groups were statistically significant, controlling for year to year differences, we used a general linear model (which is robust to the analysis of unbalanced designs and enables combining categorical and continuous variables ^18^) having the performance variable (sow mortality or pigs sold per sow) as the response, housing group as a fixed categorical predictor with three levels and year as a continuous predictor. Pairwise comparisons between factor level means were subsequently conducted with Tukey’s post-hoc test. To standardize the distribution of residuals, sow productivity values were log-transformed and mortality data were square-root arcsine transformed. Analyses were conducted using Minitab v. 21.1.1. P-values are two-tailed.

## Results and conclusions

**Figure 2.**
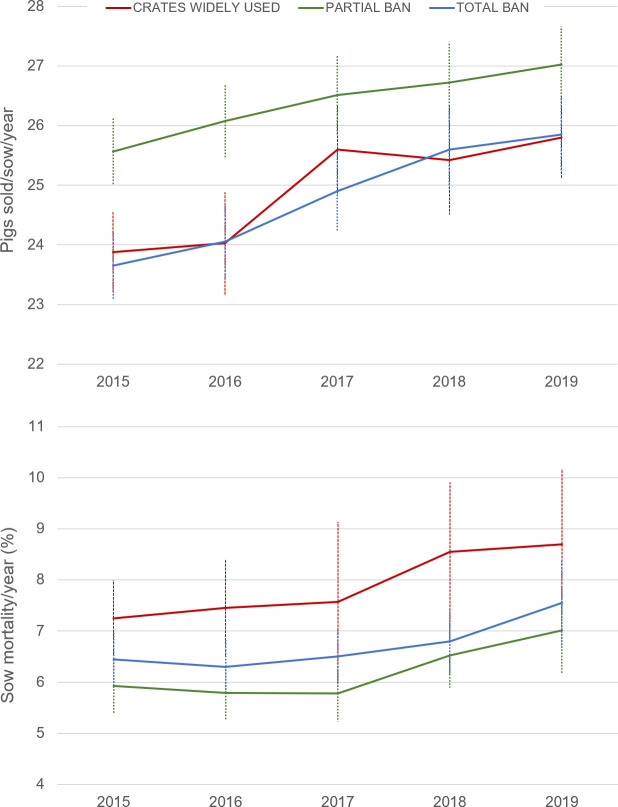
Average sow mortality (% year) and pigs sold/sow/year in three housing systems. Data from 17 countries belonging to the InterPig network, divided in three groups: (1) countries where gestation crates are the norm (Red: USA, Canada, Brazil), (2) gestation crates are restricted to (up to) the first four weeks of pregnancy (Black: Austria, Belgium, Czech Republic, Denmark, Finland, France, Germany, Hungary, Ireland, Italy, Netherlands, Spain), and (3) gestation crates are entirely banned (Blue: Sweden, United Kingdom (UK)). In the UK, data up to 2018 reflects a blend of indoor and free-range systems, and in 2019 indoor systems only.

Figure 2 shows mean values (± SEM) of sow productivity and mortality for each housing group, which have both increased over the five years ( Table 1). Figure 2 and Table 1 clearly also show that sow mortality is not greater in crate-free systems. On the contrary, significantly higher sow mortality is observed in those countries where gestation crates are still the norm (CRATE group) compared to those countries where crates have been restricted (RESTRICTED group) to four weeks after insemination (CRATE vs. RESTRICTED, *P*=0.006). Likewise, there were significant differences in the number of pigs sold per sow among the housing groups ( Table 1), with annual pig production per sow being significantly lower in countries where the use of gestation crates prevails compared to those where crates are restricted (CRATE vs. RESTRICTED, *P*=0.012). Greater productivity in RESTRICTED countries was observed despite the later weaning age (28 days or beyond) compared to CRATE countries (21 days typically).

**Table 1.** Results of general linear models testing the influence of housing groups (CRATE, RESTRICTED, BANNED) on each of the response variables (pigs sold per sow per year, and sow mortality per year) controlling for variations across years.

Factor	DF	SS	MS	F-Value	P-Value
Response: Pigs sold per sow per year (log-transformed)					
Year	1	0.009028	0.009028	9.05	0.003
Group	2	0.011947	0.005974	5.99	0.004
Error	85	0.084830	0.000998		
Total	88	0.105806			
Response: Sow mortality per year (square-root arcsine transformed)					
Year	1	0.005952	0.005952	3.34	0.071
Group	2	0.017919	0.008960	5.03	0.009
Error	85	0.151510	0.001782		
Total	88	0.175380			

These results speak against the notion that sow mortality is inherently higher, or productivity lower, in crate-free production. On the contrary, the findings presented here are in line with evidence showing that improving maternal welfare improves disease resistance, resilience and survival of piglets. ^3^^,^^4^^,^^19^^,^^20^ As observed in the transition of laying hens to cage-free systems, ^13^ variability in sow mortality might be observed during any transition from one housing system to another, though it is expected to decrease rapidly as farmers gain experience with the newly adopted systems. ^13^

Naturally, many are the factors that can influence the number of pigs sold per sow and sow mortality other than the crating of pregnant mothers. They include differences in environmental factors, genetics, nutrition, the type of feeding system, use of nursing sows, building design, farm size, and management. Given existing differences in these parameters across the country groups, it is not possible to establish a causal association between the lower mortality, or greater productivity, and restrictions on crate use. For example, all countries where crates are still the norm are in the American continent, whereas those where they were partially banned are in Europe, where factors such as genetics, management, farm sizes and climate differ. Similarly, data on the annual number of pigs sold per sow in the two countries where gestation crates were completely banned (UK and Sweden) are likely influenced by factors such as the inclusion of data for outdoor systems in the UK. The influence of factors other than crating is particularly likely given the small number of countries both in the CRATE and BANNED groups. Still, the very fact that factors other than housing can have a greater influence on the technical-economic results of sows challenges the claims investigated, of a necessary negative effect of loose housing on sow mortality and performance.

It is important to highlight that mortality and productivity are not necessarily good indicators of welfare. Although higher death rates can indicate poorer health in morbid animals, mortality fails to capture the impact of non-fatal outcomes of disease, injury and deprivations on welfare (what makes animals suffer is not necessarily what kills them). ^13^ There is also no necessary correlation between welfare and productivity. Management and genetic selection for higher productivity can in fact be linked with a higher likelihood of behavioral disorders and production diseases (diseases that become more prevalent or severe in proportion to the potential productivity of the system). ^21^ For example, hyperprolific sows often experience a higher incidence of farrowing complications, such as postpartum dysgalactia and retention of placenta. ^14^ In fact, it is precisely because the interests of animals and the economic needs of producers can be out of line that societal pressure for improved welfare standards is needed.

Changes towards crate-free housing are currently underway in many countries and affect millions of pigs annually. The present findings should be considered to guide debate on policies and legislation affecting the welfare of breeding pigs.

## Data availability

### Underlying data

Open Science Framework: Productivity of mother pigs is lower in countries that still confine them in gestation crates. https://doi.org/10.17605/OSF.IO/G4DK2 ^17^

This project contains the following underlying data:

• DataSowMortalityProductivity.xlsx (Data on sow mortality and pigs sold per sow per year, from 2015 to 2019, for 17 countries in the InterPig Network)

Data are available under the terms of the Creative Commons Attribution 4.0 International license (CC-BY 4.0).
