# Supplementary material for: Productivity of mother pigs is lower, and mortality greater, in countries that still confine them in gestation crates
Source: F1000Res. 2022 Aug 4;11:564. Originally published 2022 May 24. [Version 2] doi: 10.12688/f1000research.122042.2 (PMC9379333; doi:10.12688/f1000research.122042.2)
Supplement: Peer review history

# Peer review: Reviewer response for version 2

**DOI:** 10.5256/f1000research.137001.r146508

**Published:** 2022-08-15

**Authors:** Keelin O’Driscoll

**Affiliations:** 1 Teagasc Animal and Grassland Research and Innovation Centre, Moorepark, Ireland

The authors have dealt well with all the suggestions and comments by the reviewers, and I have nothing further to add.

Is the work clearly and accurately presented and does it cite the current literature?

Partly

If applicable, is the statistical analysis and its interpretation appropriate?

Partly

Are all the source data underlying the results available to ensure full reproducibility?

Yes

Is the study design appropriate and is the work technically sound?

Yes

Are the conclusions drawn adequately supported by the results?

Partly

Are sufficient details of methods and analysis provided to allow replication by others?

Partly

Reviewer Expertise:

Animal welfare, pig performance and management

I confirm that I have read this submission and believe that I have an appropriate level of expertise to confirm that it is of an acceptable scientific standard.

# Peer review: Reviewer response for version 2

**DOI:** 10.5256/f1000research.137001.r146507

**Published:** 2022-08-04

**Authors:** Elodie Merlot

**Affiliations:** 1 INRAE, PEGASE, Saint-Gilles, France

The authors have correctly responded to my concerns. I have no further comments regarding this manuscript.

Is the work clearly and accurately presented and does it cite the current literature?

Partly

If applicable, is the statistical analysis and its interpretation appropriate?

No

Are all the source data underlying the results available to ensure full reproducibility?

Yes

Is the study design appropriate and is the work technically sound?

Partly

Are the conclusions drawn adequately supported by the results?

Partly

Are sufficient details of methods and analysis provided to allow replication by others?

Yes

Reviewer Expertise:

I am a researcher in the field of animal sciences at the National Research Institute for Agriculture, Food and Environment (INRAE), France. I have published more than 50 scientific papers on the evaluation, using physiological and behavioral tools, of the effects of various husbandry practices and housing systems on the health and welfare of pigs.

I confirm that I have read this submission and believe that I have an appropriate level of expertise to confirm that it is of an acceptable scientific standard.

# Peer review: Reviewer response for version 1

**DOI:** 10.5256/f1000research.133979.r142491

**Published:** 2022-07-12

**Authors:** Keelin O’Driscoll

**Affiliations:** 1 Teagasc Animal and Grassland Research and Innovation Centre, Moorepark, Ireland

**General comments**

 I understand that this is a brief report, but there should be reference somewhere to the confounding factors and limitations of the results, as requested in the instructions for authors. For starters, there are only 2 countries in the ‘total ban’ category, and both of these have systems that differ significantly from those typically used in the other categories (e.g. lots of outdoor sows in the UK, Sweden requires nesting material, ban on farrowing crates etc.). It is misleading not to mention these other significant system differences, as well as the fact there is no mention of the possibility of the effect of typical genetics of the countries in the various categories.

 The description of the statistical analysis and outputs are not written clearly. I suggest the authors consult with a statistical colleague/expert to help them with this. Details below.

**Abstract**

- In the abstract, there is not enough information in the methods or information either as to which years of data were used.

**Introduction**

- In the second paragraph, it would be useful to state that gestation crates are already banned in the EU (after 28 days), not just that there is pressure from the public to ban crates in the future.
- In the final paragraph, I think it is valid to explore the claims even from the perspective of actual performance, as well as welfare – if the producers in countries where gestation crates are asking to weigh up the performance aspects of sows relative to their welfare, it is important to determine whether their claims re performance are actually valid (as well as using mortality and performance as a proxy for welfare).

**Materials and methods**

- First paragraph: I understand your meaning, but to people not familiar with Interpig data the impression is that it is primarily used for sow metrics, whereas it is much more extensive than that.
- Second paragraph. State the years you used in the first sentence, as it will make the paragraph more concise. Why did you not use data from 2020, or prior to 2015?
- I don’t understand why you used data from AHDB (Agriculture and Horticulture Development Board) and EMBRAPA (Brazilian Agricultural Research Corporation) when you state earlier that you are using Interpig data. Why use these sources as well?
- Figure 1. I suggest you use ‘a’, ‘b’, ‘c’ for the different aspects of the figure (e.g. ‘a’ for the pie chart) to make it a bit easier to follow.
- Fourth paragraph: Why do you state you "We *‘also’* investigated the extent to which potential differences in sow mortality and productivity among housing groups were statistically significant" How does this differ to what is described in the first paragraph?
- Year should not be a covariate as there are less than 6 levels, it would be better treated as a fixed effect. Tukeys is an adjustment of calculated p-values, not a test in its own right.

**Results and conclusions**

- Page 4: Evidently year was not included as a covariate, because if it was then there would be no lsmeans for each year for each category.  In L2 of the results, I don’t understand what the 2 different tests results refer to – one is the effect of year, what is the other one?
- Page 5:
  - Line 1: There is no need to include ‘GLM’ in the parentheses, as you have already stated that is the test used.
  - Line 3: Which pair-wise comparison are you referring to – is it where crates are the norm, vs where crates are allowed partially? Same for the last sentence in the paragraph. There is no need to refer to ‘Tukeys test’ as it is not a test. You could instead label the categories more concisely (e.g. USED, PARTIAL, BANNED) and then state clearly which comparison you are referring to in the text, followed by the P-value in the parentheses.

Is the work clearly and accurately presented and does it cite the current literature?

Partly

If applicable, is the statistical analysis and its interpretation appropriate?

Partly

Are all the source data underlying the results available to ensure full reproducibility?

Yes

Is the study design appropriate and is the work technically sound?

Yes

Are the conclusions drawn adequately supported by the results?

Partly

Are sufficient details of methods and analysis provided to allow replication by others?

Partly

Reviewer Expertise:

Animal welfare, pig performance and management

I confirm that I have read this submission and believe that I have an appropriate level of expertise to confirm that it is of an acceptable scientific standard, however I have significant reservations, as outlined above.

# Response

**Published:** 2022-08-01

**Authors:** Cynthia Schuck-Paim

**Affiliations:** Origem Scientifica, Brazil

We would like to thank Dr. O’Driscoll for the very helpful comments and suggestions, addressed below. 

***REVIEWER:** "I understand that this is a brief report, but there should be reference somewhere to the confounding factors and limitations of the results, as requested in the instructions for authors. For starters, there are only 2 countries in the ‘total ban’ category, and both of these have systems that differ significantly from those typically used in the other categories (e.g. lots of outdoor sows in the UK, Sweden requires nesting material, ban on farrowing crates etc.). It is misleading not to mention these other significant system differences, as well as the fact there is no mention of the possibility of the effect of typical genetics of the countries in the various categories."*

**AUTHORS:** Thank you for the suggestions. We agree such a discussion was missing, and have now incorporated it in the revised version (“...many are the factors that can influence the number of pigs sold per sow and sow mortality other than the crating of pregnant mothers. They include differences in environmental factors, genetics, nutrition, the type of feeding system, building design, farm size, and management. Given existing differences in these parameters across the country groups, it is not possible to establish a causal association between the lower mortality, or greater productivity, and restrictions on crate use. For example, all countries where crates are still the norm are in the American continent, whereas those where they were partially banned are in Europe, where factors such as genetics, management, farm sizes and climate differ. Similarly, data on the annual number of pigs sold per sow in the two countries where gestation crates were completely banned (UK and Sweden) are likely influenced by factors such as the inclusion of data for outdoor systems in the UK. The influence of factors other than crating is particularly likely given the small number of countries both in the CRATE and BANNED groups.)” 

***REVIEWER:** "The description of the statistical analysis and outputs are not written clearly. I suggest the authors consult with a statistical colleague/expert to help them with this. D*etails below."

**AUTHORS:** Thank you. We have now expanded the explanation and justification for the analyses, and include a table (Table 1) with the complete outputs of the analyses.

***REVIEWER:** "In the abstract, there is not enough information in the methods or information either as to which years of data were used."*

**AUTHORS:** The abstract has been revised to include further information on the methods.

***REVIEWER:** "In the second paragraph, it would be useful to state that gestation crates are already banned in the EU (after 28 days), not just that there is pressure from the public to ban crates in the future."*

**AUTHORS:** Thanks, this information has been now incorporated into the introduction.

***REVIEWER:** "In the final paragraph, I think it is valid to explore the claims even from the perspective of actual performance, as well as welfare – if the producers in countries where gestation crates are asking to weigh up the performance aspects of sows relative to their welfare, it is important to determine whether their claims re performance are actually valid (as well as using mortality and performance as a proxy for welfare)."*

**AUTHORS:** We now clarify that we are exploring whether the specific claims made by producers are factually valid (regarding mortality and performance), that we are measuring the performance indicator of greater economic interest to producer, and address more thoroughly the extent to which mortality and performance are good proxies of welfare (“It is important to highlight that mortality and productivity are not necessarily good indicators of welfare. Although higher death rates can indicate poorer health in morbid animals, mortality fails to capture the impact of non-fatal outcomes of disease, injury and deprivations on welfare (what makes animals suffer is not necessarily what kills them) [13]. There is also no necessary correlation between welfare and productivity. Management and genetic selection for higher productivity can in fact be linked with a higher likelihood of behavioral disorders and production diseases (diseases that become more prevalent or severe in proportion to the potential productivity of the system) [20,21]. For example, hyperprolific sows often experience a higher incidence of farrowing complications, such as postpartum dysgalactia and retention of placenta [14]...”)

***REVIEWER:** "First paragraph: I understand your meaning, but to people not familiar with Interpig data the impression is that it is primarily used for sow metrics, whereas it is much more extensive than that."*

**AUTHORS:** Thank you for raising this point. We now clarify that InterPig is a “network managed by the Agriculture and Horticulture Development Board (AHDB, a board of producers and other stakeholders in the UK farming industry), which collects farm and sector data from production economists in 17 countries to provide internationally harmonized methods for meaningful comparisons of national production indicators” and, importantly, that “InterPig data are used predominantly for cost comparisons across countries”.

***REVIEWER:**  "Second paragraph. State the years you used in the first sentence, as it will make the paragraph more concise. Why did you not use data from 2020, or prior to 2015?"*

**AUTHORS:** In the revised version of this Brief Report the choice of years is justified more thoroughly, explaining that “To reflect the most recent statistics, we used the last five years of publicly available data”, and that the year 2020 was not used because, for one of the variables (sow mortality), “data for 2020 is only available for seven of the 17 countries”. Data prior to 2015 was not used since “data on the number of pigs sold per sow annually is described in the AHDB annual reports for the period 2015-2020” only.

***REVIEWER:** "I don’t understand why you used data from AHDB (Agriculture and Horticulture Development Board) and EMBRAPA (Brazilian Agricultural Research Corporation) when you state earlier that you are using Interpig data. Why use these sources as well?"*

**AUTHORS:** Thank you for the opportunity to clarify the use of sources. We now explain that InterPig is “a network managed by the Agriculture and Horticulture Development Board (AHDB)”, and that “Interpig data on the number of pigs sold per sow annually is described in the AHDB annual reports”. We also explain that “Sow mortality statistics, as collected by the Interpig network, have been made publicly available in the reports of the Brazilian Agricultural Research Corporation (a member institution)”.

***REVIEWER:**  "Figure 1. I suggest you use ‘a’, ‘b’, ‘c’ for the different aspects of the figure (e.g. ‘a’ for the pie chart) to make it a bit easier to follow."*

**AUTHORS:** Thank you, the suggestion has been incorporated into Figure 1.

***REVIEWER:** "Fourth paragraph: Why do you state you "We ‘also’ investigated the extent to which potential differences in sow mortality and productivity among housing groups were statistically significant" How does this differ to what is described in the first paragraph?"*

**AUTHORS:** Thanks for catching this inconsistency, it has been corrected now.

***REVIEWER:** "Year should not be a covariate as there are less than 6 levels, it would be better treated as a fixed effect. Tukeys is an adjustment of calculated p-values, not a test in its own right.Page 4: Evidently year was not included as a covariate, because if it was then there would be no lsmeans for each year for each category.  In L2 of the results, I don’t understand what the 2 different tests results refer to – one is the effect of year, what is the other one?"*

**AUTHORS: **Given the continuous nature of the predictor year, and its consistent effect across years, having it fit as a continuous predictor (1 degree of freedom) makes it more sensitive than fitting it as a categorical variable (Grafen). We have now included a table with the complete output of the statistical analyses, which we hope to have clarified what each test result refers to.

***REVIEWER Page 5:** "Line 1: There is no need to include ‘GLM’ in the parentheses, as you have already stated that is the test used."*

**AUTHORS:** Thank you, it has been removed.

***REVIEWER Line 3:** "Which pair-wise comparison are you referring to – is it where crates are the norm, vs where crates are allowed partially? Same for the last sentence in the paragraph. There is no need to refer to ‘Tukeys test’ as it is not a test. You could instead label the categories more concisely (e.g. USED, PARTIAL, BANNED) and then state clearly which comparison you are referring to in the text, followed by the P-value in the parentheses."*

**AUTHORS:** The groups have been relabeled accordingly, and the comparisons clarified. 

 Thank you once more for the time and detailed comments, which we hope to have addressed satisfactorily.

 Yours, 

 Cynthia Schuck-Paim and Wladimir Alonso

# Peer review: Reviewer response for version 1

**DOI:** 10.5256/f1000research.133979.r142487

**Published:** 2022-07-04

**Authors:** Elodie Merlot

**Affiliations:** 1 INRAE, PEGASE, Saint-Gilles, France

**Is the work clearly and accurately presented and does it cite the current literature?**

- The work is very clearly presented. It cites some current literature as well as documents from governmental, European or non-governmental organizations. However, besides literature dealing with the most severe health problems generated in sows, a few other articles could have been cited as well in the introduction, for example dealing with the behavioral effects of rearing loose or crated sows during gestation, which are relevant indicators to asses sow welfare. (for example, Boyle et al, 2002, Appl Anim Behav Sci 76:119-134; Jang et al, 2017, Livestock Sci 199:37-45; Zhou et al, 2014, Animal, 8: 1162-1169)

**Is the study design appropriate and does the work have academic merit?**

- This is a very brief study involving a limited amount of data, which fits with the scope of a report for F1000Research. ****It is based on the analysis of two variables (the sow mortality rate and pigs sold annually per sow), and the data have been collected by others (data are accessible freely on internet in reports edited by the Agriculture and Horticulture Development Board). However, looking at the data available in the AHDB report, I am not convinced that the two most relevant variables were chosen by authors, nor that the report would have been too long if some additional variables had been analyzed here.
- Indeed, except in cases of prenatal imprinting (which may occur here), the further away from the birth, the more the effects of gestation tend to be diluted by other life events (potential biases) that have occurred since. Thus, the number of sold pigs per sow could have been influenced by many other factors than crating the pregnant mothers, and which would have influenced the life of pigs in between. This limitation is compounded by the fact that the number of countries included in the study is unbalanced in the 3 groups that are compared and biased in terms of geographic distribution. This increases the likelihood that uncontrolled confounding factors will influence the result observed here.
- The number of weaned pigs per sow could have been studied also to enrich the study. Although it is of less economic interest than the number of pigs sold per sow, it is of greater biological interest. Indeed, the number of weaned pigs is particularly relevant because it is highly correlated with pre-weaning mortality, and most mortality in pig production occurs before weaning. Don’t you think that this 3rd variable could be added to your study?

**Are sufficient details of methods and analysis provided to allow replication by others?**

- The nature of the data used could be described more accurately. It could be specified, because it is not obvious for non specialists, that culled sows are not included in the mortality rate. Culling rate and sow longevity are unfortunately not available in the AHDB report, but this information would have been very interesting for this study.
- It should be also very briefly explained how the number of pigs sold annually per sow is calculated/standardized. In some farms/countries, pigs are raised in the same farm until slaughter age, in some others they are sold to fattening farms after weaning. These variations make the comparison between countries uneasy.
- What Interpig is needs to be explained, so that the reader can get an idea of the reliability of the data produced by this organization. It might be indicated that InterPig belongs to the Agriculture and Horticulture Development Board (AHDB), which is not a public statistic institute, but receive national data from “production economists” working in various public or private technical institutes…

**If applicable, is the statistical analysis and its interpretation appropriate?**

- The statistical result is clear. However, the results are based on the comparison between a group of 3 countries (using gestation crate), a group of 12 countries (using crates only for a few weeks at the beginning of gestation), and lastly a group of 2 countries having sows loose for all gestation. This is not an optimal design to perform a general linear Model (2 and 3 countries in groups are too small numbers for this kind of statistical model). A non-parametric test would have been more adequate, and whatever the statistical model used, the discussion must mention the limit of the small number of countries. When a group contains 2 or 3 countries, an uncontrolled factor influencing the variable of interest in one of these countries can influence dramatically the outcome of the statistical result.

**Are all the source data underlying the results available to ensure full reproducibility?**

- Yes​​​​​​

**Are the conclusions drawn adequately supported by the results?**

- European breeders now commonly recognized the absence of negative effect of loose housing on the technical-economic results of sows. Therefore, I support the conclusion of this paper, but I am not sure that the data presented here are irrefutable proof. As mentioned above, I think that the limitations and possible biases of the study should be mentioned. The grouping of countries that has been done leads to compare countries blocking or not their pregnant sows, but it compares at the same time American countries with European countries. These regions do not have the same animal genetics, the same climate, the same sources of feed, the same size of farms, etc. There are many confounding factors that can lead to an erroneous conclusion. Some of these possible biases must be cited in the discussion.
- The results for UK and Switzerland, which have fully loose sows during gestation, might be discussed in one or two lines as well.

**Other minor comments:**

- The area and size of pens and crates must be indicated in units of the universal measurement system (meters).
- In the second paragraph of the introduction, it is necessary to specify, for more clarity, that in the European Union, the individual crates are already prohibited during a period beginning four weeks after the mating and ending one week before the date planned for the farrowing.
- In the legend of figure 2, it is not clear why authors also tried to analyze the data after removing Brazil from group 1. Since they did not remove it after all, is it useful to keep this comment? If yes, please provide some more explanations.

Is the work clearly and accurately presented and does it cite the current literature?

Partly

If applicable, is the statistical analysis and its interpretation appropriate?

No

Are all the source data underlying the results available to ensure full reproducibility?

Yes

Is the study design appropriate and is the work technically sound?

Partly

Are the conclusions drawn adequately supported by the results?

Partly

Are sufficient details of methods and analysis provided to allow replication by others?

Yes

Reviewer Expertise:

I am a researcher in the field of animal sciences at the National Research Institute for Agriculture, Food and Environment (INRAE), France. I have published more than 50 scientific papers on the evaluation, using physiological and behavioral tools, of the effects of various husbandry practices and housing systems on the health and welfare of pigs.

I confirm that I have read this submission and believe that I have an appropriate level of expertise to confirm that it is of an acceptable scientific standard, however I have significant reservations, as outlined above.

## References

[1] : Effect of gestation housing on behaviour and skin lesions of sows in farrowing crates. *Applied Animal Behaviour Science* .2002;76(2) : 10.1016/S0168-1591(01)00211-8 119-134 10.1016/S0168-1591(01)00211-8

[2] : Comparing gestating sows housing between electronic sow feeding system and a conventional stall over three consecutive parities. *Livestock Science* .2017;199: 10.1016/j.livsci.2017.02.023 37-45 10.1016/j.livsci.2017.02.023

[3] : Group housing during gestation affects the behaviour of sows and the physiological indices of offspring at weaning. *Animal* .2014;8(7) : 10.1017/S1751731114001025 1162-9 10.1017/S1751731114001025 24801378

# Response

**Published:** 2022-08-01

**Authors:** Cynthia Schuck-Paim

**Affiliations:** Origem Scientifica, Brazil

We would like to thank Dr. Merlot for the insightful comments and helpful suggestions. In the revised version we have addressed all the points made, as well as thoroughly checked the Report for greater clarity.

***REVIEWER:** "The work is very clearly presented. It cites some current literature as well as documents from governmental, European or non-governmental organizations. However, besides literature dealing with the most severe health problems generated in sows, a few other articles could have been cited as well in the introduction, for example dealing with the behavioral effects of rearing loose or crated sows during gestation, which are relevant indicators to asses sow welfare. (for example, Boyle et al, 2002, Appl Anim Behav Sci 76:119-134; Jang et al, 2017, Livestock Sci 199:37-45; Zhou et al, 2014, Animal, 8: 1162-1169)"*

**AUTHORS:** We thank Dr. Merlot for the suggestions, additional references on the behavioral effects of the rearing system have been incorporated in the revised version.

***REVIEWER:** "This is a very brief study involving a limited amount of data, which fits with the scope of a report for F1000Research. It is based on the analysis of two variables (the sow mortality rate and pigs sold annually per sow), and the data have been collected by others (data are accessible freely on internet in reports edited by the Agriculture and Horticulture Development Board). However, looking at the data available in the AHDB report, I am not convinced that the two most relevant variables were chosen by authors, nor that the report would have been too long if some additional variables had been analyzed here. Indeed, except in cases of prenatal imprinting (which may occur here), the further away from the birth, the more the effects of gestation tend to be diluted by other life events (potential biases) that have occurred since. Thus, the number of sold pigs per sow could have been influenced by many other factors than crating the pregnant mothers, and which would have influenced the life of pigs in between. This limitation is compounded by the fact that the number of countries included in the study is unbalanced in the 3 groups that are compared and biased in terms of geographic distribution. This increases the likelihood that uncontrolled confounding factors will influence the result observed here.*

*The number of weaned pigs per sow could have been studied also to enrich the study. Although it is of less economic interest than the number of pigs sold per sow, it is of greater biological interest. Indeed, the number of weaned pigs is particularly relevant because it is highly correlated with pre-weaning mortality, and most mortality in pig production occurs before weaning. Don’t you think that this 3rd variable could be added to your study?"*

**AUTHORS**: Dr. Merlot is correct in that many factors other than the housing system affect performance and welfare. In the revised version we include such a discussion. 

 Regarding the choice of parameters investigated, we now justify it more clearly, explaining that in this Brief Report our main goal is to explore whether the specific claims often made by industry representatives - which concern piglet output per sow and sow mortality (e.g., “...the California Department of Food and Agriculture has considered that use of loose housing systems may lead to “lower piglet output per animal and increased breeding pig mortality”) - are factually valid. The report thus focuses on the parameter of greater interest from the producers’ perspective, examining whether their claims are justified (“the number of pigs sold annually per sow is of greater economic interest than the compounding factors cited, hence it is the parameter of choice in economic assessments of the impact of sow housing reforms”). Additionally, and although we concur with the reviewer that the number of weaned pigs per sow is a variable of biological interest, the dataset for this variable seems to be incomplete, with missing data for Canada in some of the years.

***REVIEWER: "**The nature of the data used could be described more accurately. It could be specified, because it is not obvious for non specialists, that culled sows are not included in the mortality rate. Culling rate and sow longevity are unfortunately not available in the AHDB report, but this information would have been very interesting for this study."*

**AUTHORS:** We thank Dr. Merlot for the suggestion, we now clarify this point in the revised version (“Sow mortality is represented by the percentage of sows that die on the farm during the year. The Interpig network does not provide estimates of sow culling rates”)

***REVIEWER:** "It should be also very briefly explained how the number of pigs sold annually per sow is calculated/standardized. In some farms/countries, pigs are raised in the same farm until slaughter age, in some others they are sold to fattening farms after weaning. These variations make the comparison between countries uneasy."*

**AUTHORS:** Thank you once more. The revised version now explains that “We analyzed the number of pigs sold annually per sow and sow mortality per year. The former parameter is very informative of sow productivity, being compounded by several factors: pigs born alive per litter, litters per sow per year and mortality of pigs over the production cycle [pigs sold/sow/year = pigs weaned/sow/year * ((100-rearing mortality)/100) * ((100-finishing mortality)/100), where pigs weaned/sow/year = pigs born alive per litter * litters/sow/year * ((100-pre-weaning mortality)/100)].”

***REVIEWER**: "What Interpig is needs to be explained, so that the reader can get an idea of the reliability of the data produced by this organization. It might be indicated that InterPig belongs to the Agriculture and Horticulture Development Board (AHDB), which is not a public statistic institute, but receive national data from “production economists” working in various public or private technical institutes…"*

**AUTHORS:** Indeed, such an explanation was missing. The revised version reads “We use publicly available data from InterPig, a network managed by the Agriculture and Horticulture Development Board (AHDB, a board of producers and other stakeholders in the UK farming industry), which collects farm and sector data from production economists in 17 countries to provide internationally harmonized methods for meaningful comparisons of national production indicators. ….. InterPig data are used predominantly for cost comparisons across countries…)”.

***REVIEWER:** "The statistical result is clear. However, the results are based on the comparison between a group of 3 countries (using gestation crate), a group of 12 countries (using crates only for a few weeks at the beginning of gestation), and lastly a group of 2 countries having sows loose for all gestation. This is not an optimal design to perform a general linear Model (2 and 3 countries in groups are too small numbers for this kind of statistical model). A non-parametric test would have been more adequate, and whatever the statistical model used, the discussion must mention the limit of the small number of countries. When a group contains 2 or 3 countries, an uncontrolled factor influencing the variable of interest in one of these countries can influence dramatically the outcome of the statistical result."*

**AUTHORS:** Dr. Merlot is right in that such a small sample size in two of the groups is not ideal. However, because of (1) the nature of the response variables, (2) the fact that non-parametric tests are necessarily associated with too much loss of information when data points are ranked and (3) they are unable to combine categorical (housing group) and continuous (year) predictors, their use is very limited in this case. GLM procedures have been shown to be very robust to handle unbalanced designs (Grafen & Hails, 2002. Modern Statistics for the Life Sciences. OUP). We have now included a more thorough description of the test results (Table 1). Importantly, the statistical results reflect accurately the data patterns shown in Figure 2. 

***REVIEWER:** "European breeders now commonly recognized the absence of negative effect of loose housing on the technical-economic results of sows. Therefore, I support the conclusion of this paper, but I am not sure that the data presented here are irrefutable proof. As mentioned above, I think that the limitations and possible biases of the study should be mentioned. The grouping of countries that has been done leads to compare countries blocking or not their pregnant sows, but it compares at the same time American countries with European countries. These regions do not have the same animal genetics, the same climate, the same sources of feed, the same size of farms, etc. There are many confounding factors that can lead to an erroneous conclusion. Some of these possible biases must be cited in the discussion. The results for UK and Switzerland, which have fully loose sows during gestation, might be discussed in one or two lines as well."*

**AUTHORS:** We agree that such a discussion is needed, hence include it in the revised version (“Naturally, many are the factors that can influence the number of pigs sold per sow and sow mortality other than the crating of pregnant mothers. They include differences in environmental factors, genetics, nutrition, the type of feeding system, building design, farm size, and management. Given existing differences in these parameters across the country groups, it is not possible to establish a causal association between the lower mortality, or greater productivity, and restrictions on crate use. For example, all countries where crates are still the norm are in the American continent, whereas those where they were partially banned are in Europe, where factors such as genetics, management, farm sizes and climate differ. Similarly, results for countries where gestation crates were completely banned (UK and Sweden) are likely influenced by factors such as the inclusion of data for outdoor systems in the UK and the ban of farrowing crates in Sweden. The influence of factors other than crating is particularly likely given the small number of countries both in the CRATE and BANNED groups.”)

***REVIEWER:** "The area and size of pens and crates must be indicated in units of the universal measurement system (meters)."*

**AUTHORS:** Areas and sizes are now indicated using the metric system.

***REVIEWER:** "In the second paragraph of the introduction, it is necessary to specify, for more clarity, that in the European Union, the individual crates are already prohibited during a period beginning four weeks after the mating and ending one week before the date planned for the farrowing."*

**AUTHORS: **Such a clarification has been added, thank you for the suggestion.

***REVIEWER:** "In the legend of figure 2, it is not clear why authors also tried to analyze the data after removing Brazil from group 1. Since they did not remove it after all, is it useful to keep this comment? If yes, please provide some more explanations."*

**AUTHORS:** The comment was removed, thank you for the suggestion.

 We thank Dr. Merlot for the time and relevant comments, which we hope to have addressed satisfactorily.

 Yours sincerely, 

 Cynthia Schuck-Paim and Wladimir Alonso
